# Antimicrobial-Resistant *E. coli* in Goats in Qatar: Nationwide Evidence of MDR and ESBL Occurrence

**DOI:** 10.3390/antibiotics15040325

**Published:** 2026-03-24

**Authors:** Nahla O. Eltai, Cut Salsabila Fatin, Shayma A. Osman, Hebah A. Al Khatib, Abdullah A. Shaito, Asmaa A. Al Thani, Gheyath K. Nasrallah, Hadi M. Yassine

**Affiliations:** 1 Biomedical Research Centre, QU Health, Qatar University, Doha P.O. Box 2713, Qatar; cutsalsabila.fatin@qu.edu.qa (C.S.F.);; 2 Department of Biomedical Sciences, College of Health Sciences, QU Health, Qatar University, Doha P.O. Box 2713, Qatar; gheyath.nasrallah@qu.edu.qa; 3 World Health Organization Collaborating Centre (WHO CC) for Research and Capacity Building on Emerging and Re-Emerging Zoonotic Diseases, Doha P.O. Box 2713, Qatar

**Keywords:** AMR, MDR, ESBL, goat, Qatar, One Health

## Abstract

**Background/Objectives**: Data on antimicrobial resistance (AMR) in goat-derived *E. coli* within the Gulf Cooperation Council (GCC) region remain limited, and are largely restricted to studies conducted in Saudi Arabia and the UAE, with no published reports from Qatar. This study provides the first baseline characterization of AMR and extended-spectrum β-lactamase (ESBL) profiles of *E. coli* isolated from goats in Qatar. **Methods**: A total of 280 fecal samples were collected from goats across nine locations in Qatar (140 healthy and 140 diseased goats; 12 samples did not yield *E. coli* cultures). A selective agar medium was used to isolate *E. coli*, and the isolates were subsequently confirmed using the VITEK^®^ 2 Compact system. Antimicrobial susceptibility testing was performed to determine resistance profiles, and PCR assays were used to detect ESBL-associated genes. **Results**: 268 *E. coli* isolates were recovered from 280 samples. AMR analysis revealed a high prevalence of tetracycline resistance among *E. coli* isolates (53%), consistently observed across all nine sampling locations. Ampicillin resistance was also widespread. AMR was detected in isolates from both healthy and diseased goats; however, gentamicin resistance was found exclusively in the isolates from diseased animals. Overall, 44 isolates (16%) were classified as multidrug resistant (MDR), while nine isolates (3%) demonstrated ESBL production based on cefotaxime resistance. MDR and ESBL-producing *E. coli* were detected across all nine locations and in both healthy and diseased animals, with MDR strains occurring more frequently than ESBL producers. PCR analysis identified ESBL-associated genes, namely, *bla*_CTX-M_ in nine isolates and *bl*a_TEM_ in three isolates. **Conclusions**: Goats in Qatar harbor multidrug-resistant and ESBL-producing *E. coli*, highlighting their role as AMR reservoirs within a One Health framework. The high resistance rates to commonly used antibiotics, particularly tetracycline and ampicillin, across health statuses and geographic locations suggest potential influences of local management practices and environmental factors. The detection of ESBL genes, notably *bla*_CTX-M_ and *bla*_TEM_, underscores the need for prudent antimicrobial use and the implementation of integrated One Health surveillance programs to mitigate potential public health risks and to support national AMR surveillance and antimicrobial stewardship efforts across the region.

## 1. Introduction

The escalating challenge of antimicrobial resistance (AMR) is a complex One Health problem stemming from interactions between the human, animal, and environmental sectors. The extensive use of overlapping antimicrobial classes in humans, veterinary medicine, and agriculture fosters the emergence of resistant microorganisms [1]. At the same time, excessive and often unregulated antibiotic use, particularly in livestock production, further accelerates resistance development and the spread of resistance [2,3]. Consequently, food-producing animals frequently display higher levels of AMR than those in natural habitats with limited antimicrobial exposure [1,4]. Antibiotic-resistant bacteria and resistance genes from food animals can be disseminated to humans directly through close connections [5] and indirectly via food products, wastewater, soil, and manure-enriched environments [6,7]. Additionally, gaps in surveillance and limited understanding of environmental drivers, including wastewater, sanitation, and hygiene, continue to pose significant challenges [5]. Addressing AMR has been widely recognized as requiring coordinated interventions, including strengthened surveillance and antibiotic use regulations in livestock, increased availability of vaccines, diagnostics, and high-quality medications [2,8].

*Escherichia coli* (*E. coli*) is a Gram-negative bacterium found in the intestinal tracts of humans and animals. It is commonly used as a sentinel organism for AMR monitoring, due to its high propensity to acquire and express resistance determinants [9,10]. The increasing occurrence of multidrug-resistant (MDR) *E. coli* in food-producing animals represents a growing concern for animal and public health [11,12]. Antimicrobial use in livestock has been closely linked to the emergence and spread of resistant *E. coli* strains, making resistance profiling in this species a practical and widely applied surveillance approach [11,13]. However, data on AMR from livestock, particularly small ruminants, remain limited, underscoring the need for further investigation [10,14]. AMR in *E. coli* derived from goats has emerged as a significant concern, with studies documenting high levels of resistance, including MDR, across diverse production systems and geographical regions, highlighting goats as important reservoirs of resistant bacteria [13]. The presence of such organisms in both healthy and clinically affected animals further indicates the widespread circulation of resistance determinants within goat production environments [15,16]. Poor hygiene, suboptimal management, and clinical conditions such as diarrhea have been associated with increased shedding of resistant *E. coli*, underscoring the role of environmental and health-related stressors in the emergence of AMR [17,18]. Consistent with this, small ruminant production systems, including sheep and goats, have been recognized as reservoirs of antimicrobial resistance genes (ARGs), suggesting substantial environmental persistence and the ongoing circulation of resistant determinants [19]. The presence of MDR *E. coli* in goats, therefore, represents a potential transmission to other animals and humans through direct contact, environmental exposure, or food handling, a concern amplified by resistance to critically important antibiotics used in human medicine [9,17,20].

Despite growing recognition of these risks, significant gaps persist in understanding the dynamics of dissemination and resistance in goat populations, reinforcing the need for improved surveillance, farm management, and antimicrobial stewardship [11,12,18,20]. *E. coli* isolated from livestock in the Gulf Cooperation Council (GCC) region exhibits substantial AMR, raising concerns regarding antimicrobial use in animal production systems. Studies from the United Arab Emirates (UAE) reported comparable resistance gene profiles in *E. coli* from camels, sheep, goats, and poultry, suggesting the widespread dissemination of resistance determinants across livestock species, likely driven by similar management and antimicrobial use practices [21]. In Saudi Arabia, sheep and goats have been identified as an important reservoir of MDR *E. coli*, facilitating the spread of resistance genes within and between herds [22]. Regional studies further indicate resistance to multiple therapeutic agents, including antimicrobials considered critically important in veterinary and, in some cases, human medicine [23]. Taken together, these findings confirm that a substantial AMR burden exists across livestock species in the GCC, posing significant risks to animal health, food safety, and public health under a One Health framework. The potential for resistant bacteria to disseminate from these food-producing animals to humans, via direct contact, the food chain, or environmental pathways, underscores the urgent need for integrated surveillance and antimicrobial stewardship initiatives. Despite these findings, data on AMR in goat-derived *E. coli* within the GCC remain largely confined to Saudi Arabia and the UAE, with no published information currently available for Qatar. Given the rapid expansion of the livestock sector and close human–animal–environment interactions, establishing baseline AMR data in goats is crucial for enhancing national surveillance efforts. This study, therefore, aims to characterize the AMR profiles of *E. coli* isolated from goats in Qatar, providing the first comprehensive foundational data to support surveillance initiatives, inform evidence-based antimicrobial stewardship strategies, and enable future trend analysis. At the regional level, these data can support the development of surveillance systems targeting One Health frameworks across the GCC, as well as policy development, and facilitating cross-border comparisons in AMR monitoring.

## 2. Results

### 2.1. Geographic Distribution of Goat Samples

Al Khor had the largest proportion of samples at 17.8% (50/280), while Al Rayyan had the lowest proportion at 2.9% (8/280). Regions such as Al Jemailiya, Al Shahaniya, Al Wakra, and Umm Salal showed moderate sampling intensity, each providing approximately 40 samples (≈14% each). Lower contributions were observed by Abu Nakhla, Al Ruwais, and Roudhat Alfaras, each accounting for 20 samples or fewer (≤7% per region). Of the total samples, *E. coli* was successfully isolated from 268 goats (95.7%); the remaining twelve samples did not yield *E. coli.*

### 2.2. Antibiotic Susceptibility and Phenotypic Characterization of E. coli Isolates

**Figure 1:**
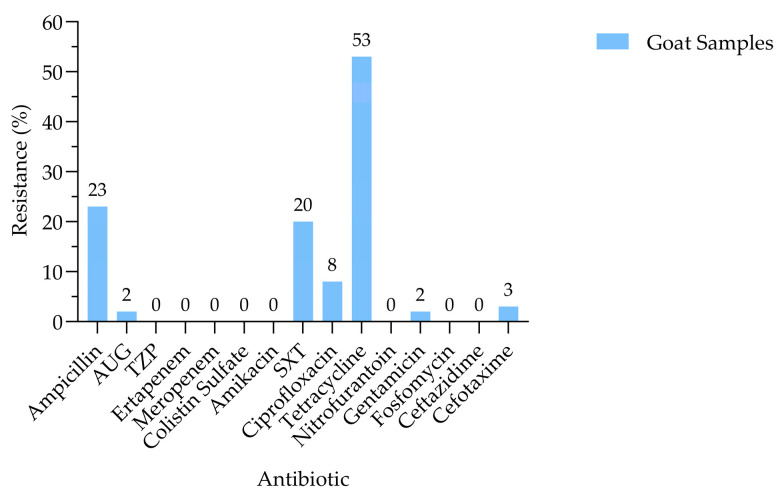
Phenotypic resistance patterns of *E. coli* isolates from goat samples (N = 268). AUG: amoxicillin–clavulanic acid, TZP: piperacillin–tazobactam, SXT: trimethoprim–sulfamethoxazole.

**Figure 2:**
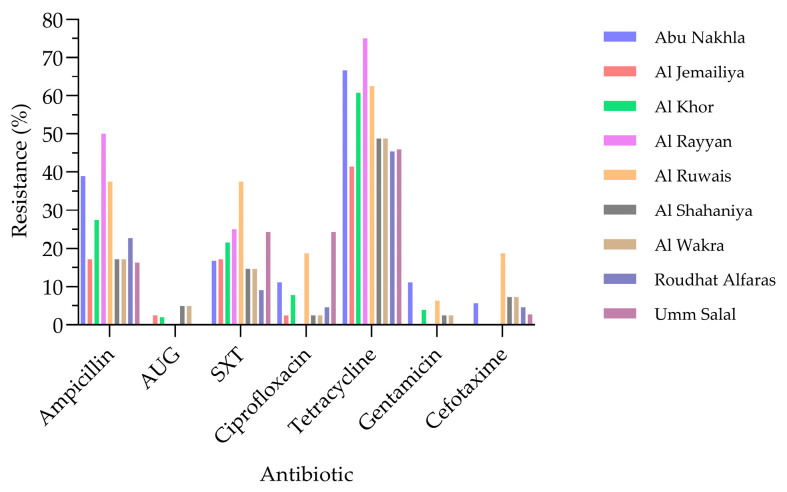
Percentage distribution of phenotypic antibiotic resistance among *E. coli* isolates from goats across locations in Qatar (N = 268). AUG: amoxicillin–clavulanic acid, SXT: trimethoprim–sulfamethoxazole.

The AMR analysis (Figure 1) revealed that tetracycline resistance was the most prevalent among the *E. coli* isolates, affecting 53% of strains. This was followed by resistance to ampicillin (23%) and trimethoprim–sulfamethoxazole (20%). Resistance to ciprofloxacin was comparatively lower (8%), yet still exceeded that observed for cefotaxime (3%), amoxicillin–clavulanic acid (2%), and gentamicin (2%). The isolates exhibited resistance to seven out of fifteen clinically relevant antibiotics tested. Antibiotic resistance patterns among *E. coli* isolates varied across geographic regions in Qatar (Figure 2). Tetracycline resistance was the most prevalent across all locations, with the highest levels observed in Al Rayyan. Ampicillin resistance was also widespread, particularly in Al Rayyan, Abu Nakhla, and Al Ruwais. Moderate resistance to trimethoprim–sulfamethoxazole (SXT) and ciprofloxacin was noted in Al Ruwais and Umm Salal, respectively. In contrast, resistance to amoxicillin–clavulanic acid (AUG), gentamicin, and cefotaxime remained consistently low across all locations.

**Figure 3:**
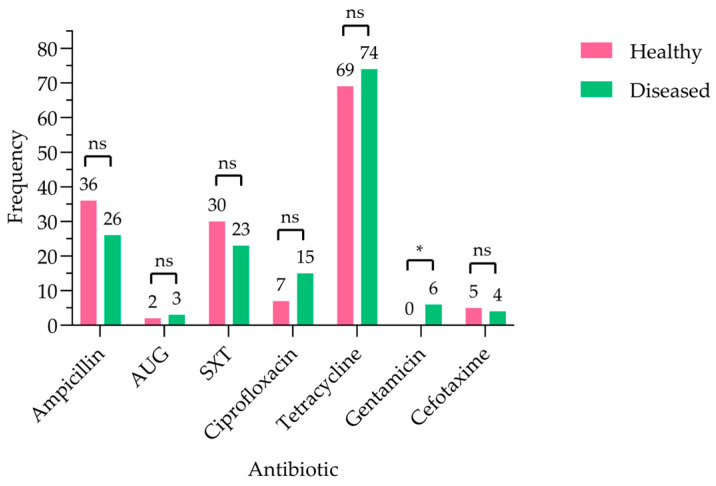
Frequency of antibiotic resistance among *E. coli* isolates obtained from healthy and diseased goats (N = 268). * indicates *p*-value is statistically significant by Fisher’s exact test (<0.0332); while ns indicates non-significance. AUG: amoxicillin–clavulanic acid, SXT: trimethoprim–sulfamethoxazole.

Distinct frequencies of antibiotic resistance were observed in *E. coli* isolates from healthy versus diseased goats (Figure 3). Tetracycline resistance was the most prevalent in both groups, observed in 69 and 74 isolates from healthy and diseased goats, respectively. Ampicillin resistance was the next most common, affecting 36 isolates from healthy goats and 26 from diseased goats. Resistance to SXT was detected in 30 healthy and 23 diseased isolates. Ciprofloxacin resistance was higher among isolates from diseased goats (N = 15) compared to those from healthy ones (N = 7). Resistance to AUG and cefotaxime remained low in both groups. While most variations in resistance frequencies between healthy and diseased goats were not statistically significant, gentamicin resistance was detected exclusively in isolates from diseased goats (N = 7) and absent in healthy goats, representing a significant difference (*p* < 0.0332). Among these cases, the reported clinical conditions were varied and included respiratory symptoms (n = 2, illness not further specified), pasteurellosis (n = 1), enteritis (n = 1), and pneumonia (n = 2), suggesting that gentamicin resistance was not associated with a single specific disease condition in this study.

**Figure 4:**
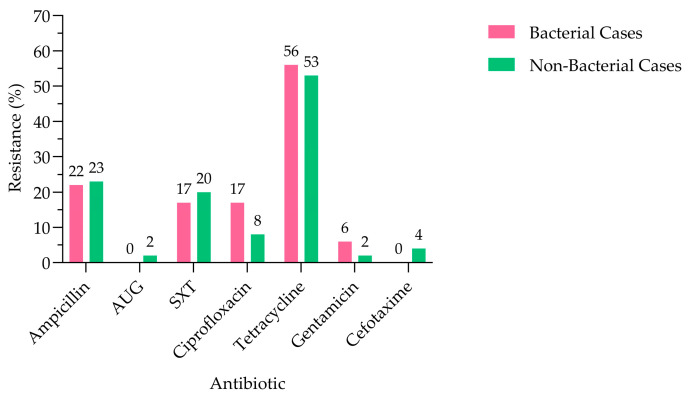
Antibiotic resistance patterns of *E. coli* isolates in primary bacterial and non-bacterial cases (N = 268). AUG: amoxicillin–clavulanic acid, SXT: trimethoprim–sulfamethoxazole.

Among the 132 diseased goats from which *E. coli* isolates were obtained, clinical symptoms were classified to differentiate primary bacterial pathologies from non-bacterial diseases. Confirmed primary bacterial infections totaled 18 cases, including enteritis (n = 6), bacterial diarrhea (n = 5), mastitis (n = 2), metritis (n = 2), John’s disease (n = 2), and abscess (n = 1). Non-bacterial conditions were predominant (n = 68) and mainly involved parasitic infections such as blood parasites (n = 13), unspecified parasitic infections (n = 12), mange (n = 4), coccidiosis (n = 3), and cryptosporidiosis (n = 1). Additional non-infectious or unspecified conditions included malnutrition (n = 5), arthritis (n = 1), abortion (n = 1), and unspecified non-respiratory illness (n = 28). The remaining 46 cases corresponded to respiratory conditions of indeterminate etiology, including unspecified respiratory illness (n = 25), pneumonia (n = 14), rhinitis (n = 4), bronchitis (n = 1), and bronchopneumonia (n = 2), which may arise from bacterial, viral, or mixed infections, thus cannot be conclusively categorized as primary bacterial diseases. Comparison of the antimicrobial resistance patterns between primary bacterial and non-bacterial cases showed similar resistance rates across most antibiotics (Figure 4). Resistance to tetracycline was the highest in both groups (56% in bacterial cases vs. 53% in non-bacterial cases), followed by ampicillin (22% vs. 23%) and trimethoprim–sulfamethoxazole (17% vs. 20%). Ciprofloxacin resistance was higher in bacterial cases (17%) compared with non-bacterial cases (8%), whereas cefotaxime resistance was detected only in the non-bacterial group (4%). Low resistance levels were observed for gentamicin (6% vs. 2%) and amoxicillin–clavulanic acid (0% vs. 2%). However, Fisher’s exact test indicated that none of the observed differences in resistance rates between bacterial and non-bacterial cases were statistically significant (*p* > 0.05).

**Figure 5:**
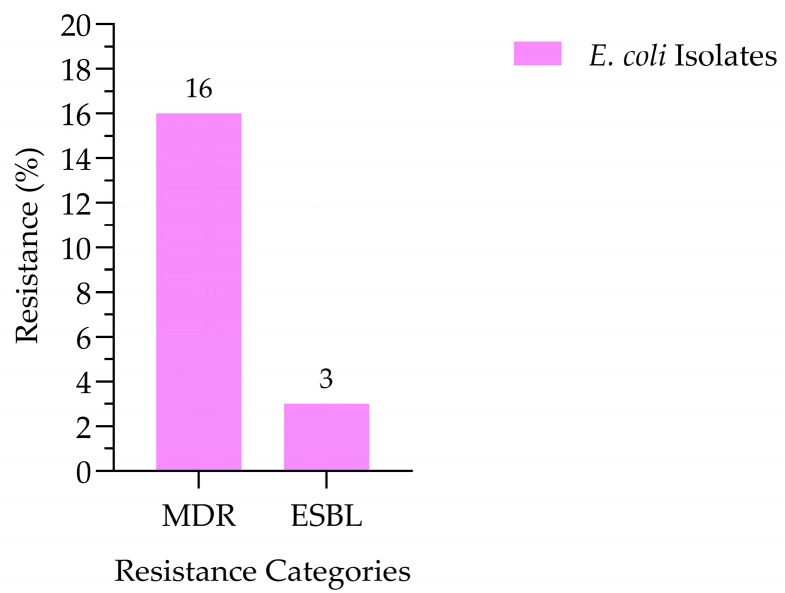
Percentage of MDR and ESBL-producing *E. coli* from goat samples (N = 268).

**Figure 6:**
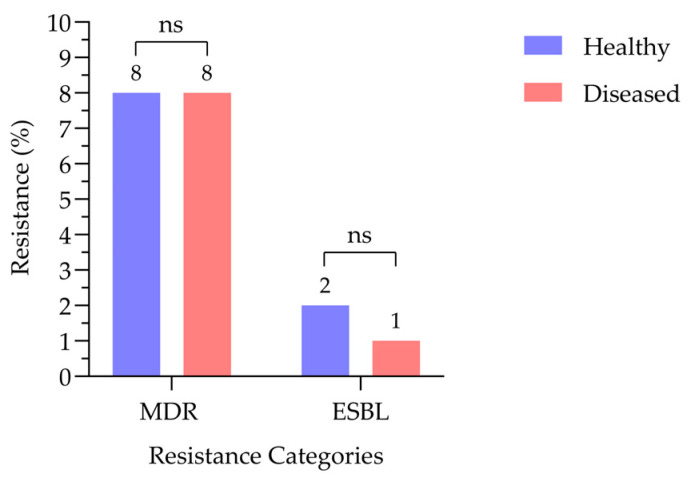
Frequency of MDR and ESBL-producing *E. coli* from healthy and diseased goat samples (N = 268). ns indicates non-significance by Fisher’s exact test.

Overall, 44 isolates (16%) were classified as MDR, exhibiting resistance to three or more antibiotic classes. Among these isolates, nine (3%) were identified as ESBL producers, based on resistance to cefotaxime. MDR and ESBL-producing *E. coli* were detected in healthy and diseased goat samples (Figure 5). The prevalence of MDR *E. coli* was comparable between healthy and diseased goats, with both groups exhibiting an identical resistance rate of 8%. ESBL-producing isolates were detected at low frequencies in both groups, accounting for 2% of healthy goats and 1% of diseased goats. Statistical analysis revealed no significant differences in MDR or ESBL occurrence between healthy and diseased animals (*p* > 0.05), indicating that the carriage of these resistant strains was not significantly associated with the animals’ clinical status (Figure 6).

**Figure 7:**
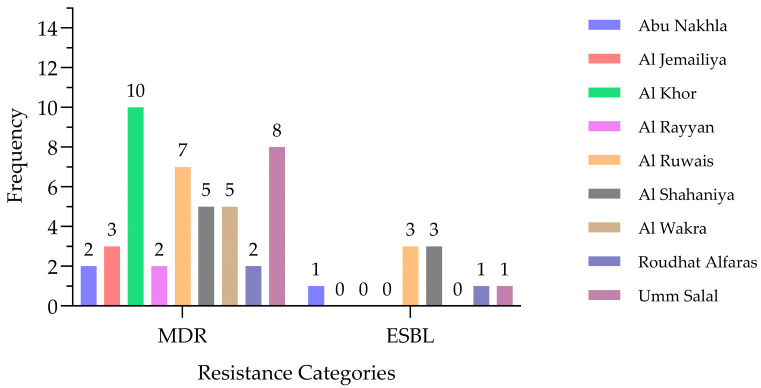
Frequency of MDR and ESBL-producing *E. coli* from goat samples across different locations in Qatar (N = 268).

MDR and ESBL-producing *E. coli* isolates were detected across all nine sampling locations in Qatar. MDR isolates were more frequently observed than ESBL producers (Figure 7). Al Khor recorded the highest number of MDR isolates (N = 10), followed by Umm Salal (N = 8), Al Ruwais (N = 7), Al Shahaniya (N = 5), and Al Wakra (N = 5). Lower MDR frequencies were identified in Al Jemailiya (N = 3), Abu Nakhla (N = 2), Al Rayyan (N = 2), and Roudhat Alfaras (N = 2). In contrast, ESBL-producing isolates were uncommon, with only three cases detected in Al Ruwais and Al Shahaniya, and single isolates identified in Abu Nakhla, Roudhat Alfaras, and Umm Salal. An overall comparison using Fisher’s exact test for the contingency table did not reveal a statistically significant association between the sampling location and the prevalence of MDR isolates (*p* ≥ 0.05). Similarly, no statistically significant variation in the distribution of ESBL-producing isolates was observed across the sampling locations (*p* ≥ 0.05).

**Figure 8:**
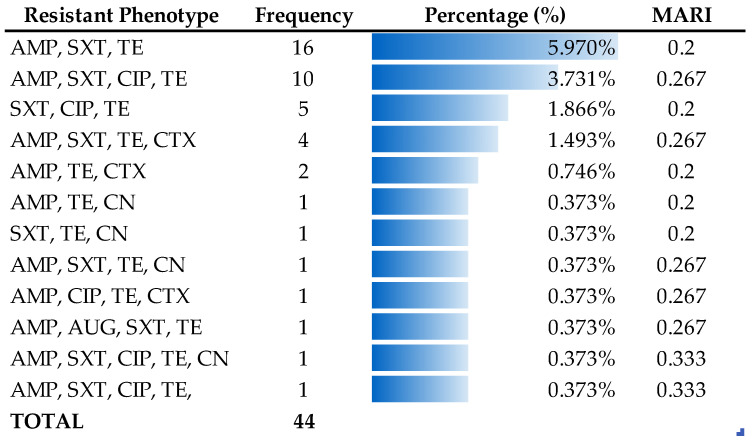
Phenotypic multidrug-resistant (MDR) resistance profile of *E. coli* isolates from goat rectal swab and fecal samples (N = 268). MARI: Multiple Antibiotic Resistance Index, AMP: ampicillin, SXT: trimethoprim–sulfamethoxazole, TE: tetracycline, CIP: ciprofloxacin, CTX: cefotaxime, CN: gentamicin, AUG: amoxicillin–clavulanic acid.

MDR patterns in *E. coli* isolates were evaluated using the multiple antibiotic resistance index (MARI). MARI values ≤ 0.2 were classified as low-risk contamination, whereas values > 0.2 were classified as high-risk contamination (Figure 8). Some MDR phenotypes were observed only once among the isolates (n = 1), indicating that these resistance patterns were rare occurrences.

### 2.3. Molecular Detection of ESBL-Encoding Genes in E. coli Isolates from Goat Samples

The *bla*_CTX-M_ and *bla*_TEM_ genes, both associated with ESBL production, were detected among *E. coli* isolates exhibiting cefotaxime resistance (Table 1). The bla_CTX-M_ gene was identified in six isolates (66.7%). The co-occurrence of *bla*_CTX-M_ and *bla*_TEM_ genes was observed in three isolates (33.3%), indicating the presence of multiple ESBL determinants in a subset of isolates. Overall, nine isolates harbored the *bla*_CTX-M_ gene, whereas three isolates carried the *bla*_TEM_ gene.

## 3. Discussion

The detection of resistance to seven of the fifteen antimicrobials tested highlights the substantial AMR burden among *E. coli* isolated from goats, reinforcing their role as potential reservoirs of resistant bacteria within the One Health continuum, where resistance can circulate between animals, humans, and the environment. Tetracycline resistance was most prevalent (53%), consistent with previous livestock studies [11,12,17], reflecting the long-standing and widespread use in food–animal production. Resistance to ampicillin (23%), trimethoprim–sulfamethoxazole (20%), and ciprofloxacin (8%) was comparable to previous reports [13,16,18], though lower than rates documented elsewhere [9,11,12,15]. Despite the low prevalence, ciprofloxacin resistance remains noteworthy given the importance of fluoroquinolones in human medicine. Resistance to cefotaxime, amoxicillin–clavulanic acid, and gentamicin was minimal (≤3%) and consistent with earlier findings [12,14,24]. All isolates were susceptible to critically important antimicrobials, including carbapenems and colistin, consistent with previous studies [13,16,24,25,26,27,28]. Analyzing AMR profiles of goats and sheep in Qatar revealed notable differences between the two species over time. In the recent goat study, resistance was observed for ampicillin (23%) and ciprofloxacin (8%), whereas the older sheep study [14] reported higher resistance, with ciprofloxacin at 69.4% and ampicillin 34%. These differences may reflect changes in antibiotic usage over time, variations in management practices, or species-specific factors. For example, sheep in traditional systems often live in larger flocks, which can facilitate the spread of resistant bacteria; on the other hand, goats are frequently kept in smaller herds, which may help limit the spread. Overall, the lower resistance rates observed in goats may indicate reduced selective pressure or differences in exposure compared with sheep, with implications for livestock management and public health under a One Health framework. Nonetheless, reports of carbapenem resistance in livestock elsewhere [10,11] emphasize the need for continued One Health-based surveillance and prudent antimicrobial use in goat production systems. Clear geographic heterogeneity in AMR was observed among *E. coli* isolates from goats in Qatar (Figure 2), with tetracycline and ampicillin resistance predominating across locations, whereas resistance to other agents remained low. Such spatial variation aligns with the evidence that AMR patterns are shaped by local antimicrobial use, environmental exposure, and ecological pressures rather than being uniformly distributed [12]. Similar geographic clustering of resistance has been reported in livestock, wildlife, and the environment [29,30,31,32], emphasizing the role of local selective pressure. Comparative studies show higher resistance in rural settings in India and South Africa [33,34], whereas a multi-country analysis reported higher resistance in urban settings [35], indicating that rural–urban classification alone does not reliably predict AMR. Regional variation has also been documented in North America, where MDR in bovine *E. coli* is linked to both antimicrobial use and production-related factors [36]. The MARI was used to evaluate the risk associated with exposure to resistant bacteria, in which values ≤ 0.2 designate low-risk contamination and values > 0.2 indicate high-risk contamination (Table 1). MARI values suggest increased antimicrobial selective pressure, which may support the persistence of resistant bacteria in food-producing animals and pose potential public health risks [4]. Notably, all seven identified resistance phenotypes were MDR, with MARI values greater than 0.2, which may reflect underlying antimicrobial use practices and underscores the importance of strengthened antimicrobial stewardship within regional livestock production systems. Consistent with the preceding graph, these findings highlight the interconnected human, animal, and environmental drivers of livestock-associated AMR, with geographically structured resistance in goats posing implications for veterinary, environmental, and public health in Qatar. Resistance patterns were broadly analogous between *E. coli* isolates from healthy and diseased goats, with tetracycline predominating in both groups, indicating that AMR is not confined to clinically ill animals. Gentamicin resistance, observed exclusively in diseased goats, represents the only significant difference and suggests further selection pressure associated with antibiotic use [3,22]. The detection of resistance in healthy animals indicates that livestock-associated AMR is driven by ecological, management, and environmental factors beyond direct antibiotic exposure, with clear implications for One Health transmission pathways [6,11,18]. The occurrence of ESBL-producing *E. coli* in both groups, likely influenced by β-lactam use and production practices [11,18,22], may reflect the need for judicious antimicrobial use and integrated One Health surveillance. Geographic heterogeneity in MDR and ESBL-producing *E. coli* was observed among goats in Qatar, with MDR more prevalent, particularly in Al Khor, Umm Salal, and Al Ruwais (Figure 6), likely suggesting localized management practices and antimicrobial use [11]. ESBL-producing isolates were less common and exhibited limited spatial variation; however their presence remains epidemiologically significant due to their potential for horizontal gene transfer and co-occurring multidrug resistance [11,17,37,38]. No significant differences in MDR or ESBL prevalence were detected between healthy and diseased goats, suggesting transmission of resistant strains irrespective of health status, consistent with reports showing that asymptomatic small ruminants can act as silent reservoirs of AMR [39,40]. Geographic clustering of MDR in certain regions of Qatar likely reflects local farming practices, antimicrobial use, and environmental dissemination, including factors such as animal stocking density, sanitation conditions, and manure management practices that may influence the spread of resistant bacteria in livestock systems. Even a limited presence of ESBL-producing bacteria in goats poses a risk of transmission to other animals, farm workers, and humans, highlighting the need for integrated One Health interventions. The detection of ESBL-associated genes in goat-derived *E. coli* underlines the One Health relevance of AMR at the human–animal interface. In this study, *bla*_CTX-M_ was the dominant determinant, with some isolates also carrying *bla*_TEM_, indicate the co-occurring resistance mechanisms that facilitate horizontal gene transfer [10,38]. These findings align with regional and international reports showing that ESBL carriage in livestock is common, often coexists with MDR, and poses a risk for transmission along the food–animal–human continuum [10,38]. Despite the limited number of isolates, the presence of multiple ESBL genes, including *bla*_CTX-M_ and *bla*_TEM_, reinforces the role of farm animals as reservoirs of ESBL-producing bacteria with the potential for spillover to humans and the environment, underscoring the need for integrated One Health surveillance and prudent antimicrobial use to limit silent dissemination. These findings provide critical locally derived evidence to inform Qatar’s National Action Plan on AMR. Specifically, our results support the need to strengthen antimicrobial stewardship in veterinary practice by encouraging evidence-based prescribing over empirical treatment. Furthermore, given the interconnected food supply systems across the GCC, establishing a coordinated One Health-based surveillance program for AMR in food-producing animals is essential for regional food safety and public health. This study has some limitations. The scope did not include detailed farm management data collection, which prevented risk factor analysis of AMR. Furthermore, the specific localization of resistance genes (e.g., on plasmids) was not determined. Therefore, a key direction for future research is to use a One Health approach, employing Whole Genome Sequencing (WGS), to investigate transmission dynamics by comparing isolates from goats, humans, and environmental samples.

## 4. Materials and Methods

### 4.1. Sample Collection

**Figure 9:**
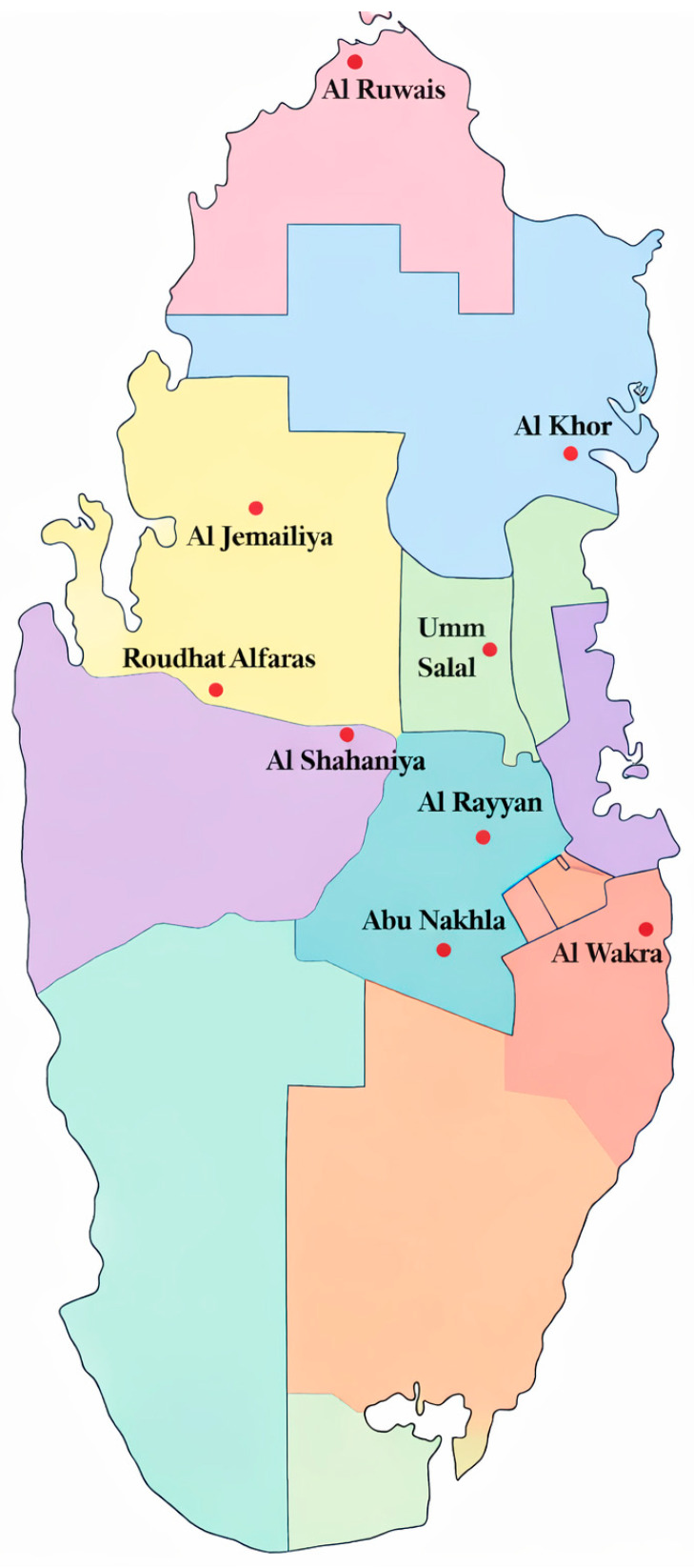
Map of Qatar showing the geographic distribution of goat sampling locations across the nine study regions.

In a cross-sectional study conducted between 4 January and 5 December 2024, fecal samples were collected from goats across all nine administrative regions of Qatar, namely Abu Nakhla, Al Khor, Al Rayyan, Al Ruwais, Al Shahaniya, Al Wakra, Al Jemailiya, Roudhat Alfaras, and Umm Salal (Figure 9). A stratified random sampling strategy was employed to ensure national representation. The number of samples collected from each region was proportional to the estimated density of the goat population in that area, and individual farms within these regions were randomly selected to capture a diverse range of management practices. All sampling procedures were conducted in accordance with institutional biosafety and ethical guidelines and were approved by the Institutional Biosafety Committee (IBC) of Qatar University, under approval number IBC-1949153-2. A total of 280 samples were collected from goats of various ages and production systems, categorized by clinical status. Specifically, 140 samples were collected from clinically healthy animals, while the remaining 140 samples were obtained from animals presenting clinical signs or diagnosed with health conditions. Reported conditions among the diseased animals included abortion, abscess, arthritis, bacterial infections, bronchitis, bronchopneumonia, coccidiosis, cryptosporidiosis, diarrhea, enteritis, mastitis, mange, malnutrition, metritis, parasitic infections, pneumonia, pasteurellosis, and rhinitis. Fecal samples were collected directly from the rectum or immediately after defecation using sterile techniques. Samples were placed in sterile containers, labeled, and transported to the laboratory on ice. Upon arrival, the fecal samples were homogenized in sterile phosphate-buffered saline (PBS) supplemented with glycerol and stored at −80 °C until subsequent microbiological and molecular analyses.

### 4.2. E. coli Isolation and Identification

First, 10 µL of the bacterial suspension was streaked onto HiCrome^TM^ *E. coli* Agar (Hi-Media, Thane, Maharashtra, India) and incubated at 37 °C for 18–24 h. Colonies exhibiting the characteristic blue-green coloration with smooth morphology, indicative of *E. coli*, were subcultured onto Nutrient Agar (Hi-Media, Maharashtra, India). Further confirmation of species identification was performed using the automated VITEK^®^ 2 Compact system (BioMérieux, Mumbai, France). The analysis, performed using the GN identification card, confirmed that all isolates were E. coli, with the system assigning an excellent level of identification confidence.

### 4.3. Antibiotic Susceptibility Testing (AST)

*E. coli* isolates were tested for susceptibility to 15 clinically relevant antibiotics (Table 2). Susceptibility to 14 of these antibiotics was determined using the standard Kirby–Bauer disk diffusion method, following M100 CLSI 2020 guidelines [40], with disks sourced from Liofilchem (Roseto degli Abruzzi, Italy). As an exception, colistin susceptibility was determined separately by the broth microdilution method using the SensiTest Colistin kit (Liofilchem, Roseto degli Abruzzi, Italy), following the manufacturer’s and CLSI recommendations [40]. AST was performed on all *E. coli* isolates recovered from 268 of the 280 total goat fecal samples. Quality control for AST was performed using *E. coli* ATCC 25922 according to CLSI guidelines. All inhibition zone diameters and MIC values fell within the recommended CLSI M100 quality control ranges.

### 4.4. Double Disk Synergy Test (DDST)

*E. coli* isolates exhibiting resistance to third-generation cephalosporins, specifically cefotaxime, defined by a zone diameter of ≤22 mm based on AST, were further screened for ESBL production using the Double Disk Synergy Test (DDST) [40,41].

Overnight cultures were adjusted to a 0.5 McFarland standard in PBS (Oxoid, UK) and inoculated onto Mueller–Hinton II Agar (MHA; Liofilchem, Italy). Amoxicillin–clavulanate (30 μg; Liofilchem, Italy) was placed at the center of each plate with cefotaxime (30 μg) and ceftazidime (30 μg) disks positioned 15 mm apart (edge to edge) on either side, and a cefoxitin disk (30 μg) was placed in any available space on the plate; the plate was then incubated at 37 °C for 18–24 h.

**Figure 10:**
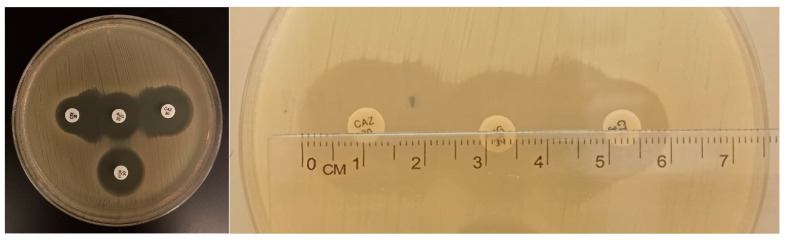
Double Disk Synergy Test (DDST) confirming ESBL-producing *E. coli* isolates obtained from goat samples using amoxicillin–clavulanic acid (30 µg), ceftazidime (30 µg), cefotaxime (30 µg), and cefoxitin (30 µg).

Isolates displaying a ≥5 mm increase in the inhibition zone of either cefotaxime or ceftazidime toward the amoxicillin–clavulanate disk, while remaining susceptible to cefoxitin, were interpreted as ESBL producers (Figure 10).

### 4.5. DNA Extraction and Polymerase Chain Reaction (PCR)

Phenotypically confirmed ESBL-producing *E. coli* isolates were screened for *bla*_CTX-M_, *bla*_SHV_, and *bla*_TEM_ genes. Genomic DNA was extracted from overnight cultures using the QIAamp UCP Pathogen Mini Kit (Qiagen, Hilden, Germany), following the manufacturer’s instructions. The extracted DNA was subjected to PCR amplification with gene-specific primers (Integrated DNA Technologies, Coralville, IA, USA), as listed in Table 3. PCR reactions were prepared in a 20 μL final volume containing 10 μL of HotStarTaq Plus Master Mix, 2 μL of RNase-free water, 0.5 μL each of forward and reverse primers designed for each target gene, 4 μL of CoralLoad Concentrate, and 3 μL of template DNA. Amplifications were carried out using the Analytik Jena Biometra TAdvanced Thermal Cycler under gene-specific cycling conditions.

For *bla*_CTX-M_, there was 1 cycle of denaturation at 95 °C for 5 min, followed by 30 cycles of denaturation at 94 °C for 25 s, annealing at 52 °C for 40 s, and elongation at 72 °C for 50 s, with a final cycle of elongation at 72 °C for 10 min. For *bla*_SHV_, there was 1 cycle of denaturation at 95 °C for 5 min, followed by 32 cycles of denaturation at 96 °C for 30 s, annealing at 58 °C for 45 s, and elongation at 72 °C for 60 s, with a final cycle of elongation at 72 °C for 10 min. For *bla*_TEM_, there was 1 cycle of denaturation at 95 °C for 5 min, followed by 32 cycles of denaturation at 96 °C for 30 s, annealing at 44 °C for 45 s, and elongation at 72 °C for 60 s, with a final elongation cycle at 72 °C for 10 min. PCR amplicons were resolved on 1% agarose gels stained with ethidium bromide (Appendix A) and visualized using an iBright CL1000 imaging system (Invitrogen; Thermo Fisher Scientific, Waltham, MA, USA), with a 100 bp Plus DNA ladder (Qiagen, Germany) used as a molecular size marker.

### 4.6. Data Analysis

Phenotypic antibiotic susceptibility and demographic data were entered into Microsoft Excel 2016 for descriptive analysis. Percent resistance, resistance patterns among *E. coli* from healthy and diseased goats, and the prevalence of MDR and ESBL production were evaluated across the sampled regions.

All graphical representations were generated using GraphPad Prism (Version 10.4.1). Statistical analyses were performed using Fisher’s exact test to compare the frequency of antibiotic resistance between *E. coli* isolates from healthy and diseased goats and to examine the distribution of MDR and ESBL-producing isolates across sampling sites in Qatar. A 95% confidence interval was applied, and *p*-values < 0.05 were considered statistically significant.

## 5. Conclusions

This study reveals that Qatar’s goat population harbors a significant reservoir of antimicrobial resistance (AMR), including high rates of resistance to important antibiotics such as tetracycline and ampicillin. The detection of mobile ESBL genes, such as *bla*_CTX-M_, underscores the clear potential for this reservoir to contribute to the broader public health challenge of AMR through horizontal gene transfer. Therefore, our findings provide foundational evidence to justify establishing a national surveillance program for antimicrobial use (AMU) in veterinary practice. Integrating such AMU data with ongoing AMR monitoring is a cornerstone of an effective One Health surveillance strategy, essential for developing evidence-based stewardship policies and protecting animal and human health in Qatar.

## Figures and Tables

**Table 1:** Detection of genes associated with ESBL production in cefotaxime-resistant *E. coli* isolates (N = 9). Categories represent mutually exclusive gene profiles: *bla*_CTX-M_ only or *bla*_CTX-M_ and bla_TEM_.

Gene Combinations	Frequency	Percentage (%)
***bla***_CTX-M_	6	66.7%
***bla***_CTX-M_, ***bla***_TEM_	3	33.3

**Table 2:** Antibiotics tested, disk concentrations, and CLSI (2020) zone diameter interpretive criteria for *E. coli*.

No.	Antibiotic	Antibiotic Class	Concentration	CLSI Susceptibility Range (mm) [40]
**1**	Ampicillin (AMP)	Penicillin	10 μg	≥17 S/R 13≤
**2**	Amoxicillin–Clavulanic Acid (AUG)	Penicillin	30 μg	≥18 S/R 13≤
**3**	Piperacillin–Tazobactam (TZP)	Penicillin–Beta-Lactamase Inhibitor	25 μg	≥21 S/R 17≤
**4**	Ertapenem (ETP)	Carbapenem	10 μg	≥22 S/R 18≤
**5**	Meropenem (MRP)	Carbapenem	10 μg	≥23 S/R 19≤
**6**	Amikacin (AK)	Aminoglycoside	30 μg	≥17 S/R 16≤
**7**	Gentamicin (CN)	Aminoglycoside	10 μg	≥15 S/R 12≤
**8**	Fosfomycin (FOS)	Phosphoric Acid Derivative	200 μg	≥16 S/R 12≤
**9**	Trimethoprim–Sulfamethoxazole (SXT)	Sulfonamide	25 μg	≥16 S/R 10≤
**10**	Ciprofloxacin (CIP)	Fluoroquinolone	5 μg	≥21 S/R 15≤
**11**	Cefotaxime (CTX)	Cephalosporin	30 μg	≥26 S/R 22≤
**12**	Ceftazidime (CAZ)	Cephalosporin	30 μg	≥21 S/R 17≤
**13**	Nitrofurantoin(F)	Nitrofuran	300 μg	≥17 S/R 14≤
**14**	Tetracycline (TE)	Tetracycline	30 μg	≥15 S/R 11≤
**15**	Colistin (Broth Microdilution)	Polymyxin	0.25–15 mg/mL	≤1 S/R 4≥

**Table 3:** Primers used for PCR amplification.

Target Gene	Primer	Sequence (5′-3′)	Amplicon Size	Reference
***bla***_TEM_	Forward Reverse	AAAATTCTTGAAGACG TTACCAATGCTTAATCA	1080 bp	[42]
***bla***_SHV_	Forward Reverse	GGGTTATTCTTATTTGTCGCT TAGCGTTGCCAGTGCTCG	929 bp	[42]
***bla***_CTX-M_	Forward Reverse	TTTGCGATGTGCAGTACCAGTAA CGATATCGTTGGTGGTGCCATA	544 bp	[42]

**Figure A1:**
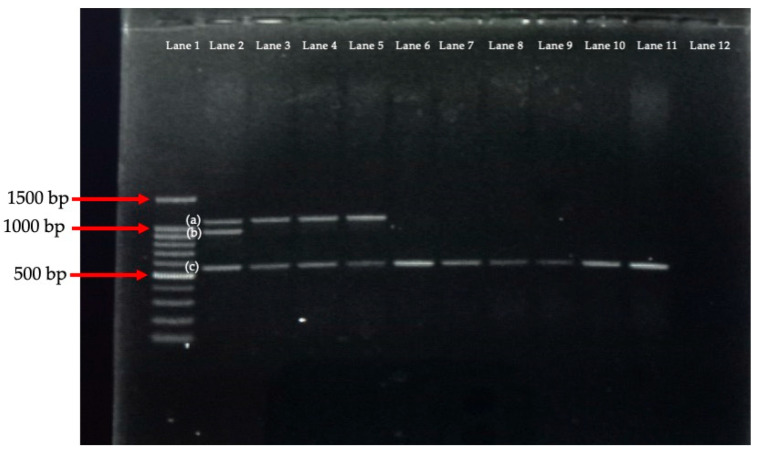
Detection of *bla*_TEM_ and *bla*_CTX-M_ genes in cefotaxime-resistant Escherichia coli isolates by PCR followed by agarose gel electrophoresis. Lane 1: 100 bp DNA ladder. Lane 2: positive controls. (a) *E. coli* NCTC 13351 carrying *bla*_TEM_ (1080 bp), (b) *E. coli* NCTC 13368 carrying *bla*_SHV_ (929 bp), and (c) *E. coli* NCTC 13462 carrying *bla*_TEM_ and *bla*_CTX- M_ (544 bp). Lanes 3–11: *E. coli* isolates showing amplification of *bla*_CTX-M_ alone (544 bp) or co-amplification of *bla*_TEM_ and *bla*_CTX-M_ (544 bp and 1080 bp). Lane 12: negative control (*E. coli* ATCC 25922).

## Data Availability

All data are included in this study in the manuscript.

## Abbreviations

The following abbreviations are used in this manuscript:

AMR	Antimicrobial Resistance
MDR	Multidrug Resistance
ESBL	Extended-Spectrum Beta-Lactamase
GCC	Gulf Cooperation Council
DDST	Double Disk Synergy Test
PCR	Polymerase Chain Reaction

## Appendix A

The detection of *bla*_TEM_ and *bla*_CTX-M_ genes in cefotaxime-resistant *E. coli* isolates was performed by PCR amplification. A 100 bp DNA ladder was used as a molecular size reference to estimate the fragment sizes. The positive controls produced distinct bands at approximately 1080 bp (*bla*_TEM_), 929 bp (*bla*_SHV_), and 544 bp (*bla*_CTX-M_), correspond5 showed amplification at approximately 1080 bp and 544 bp, indicating the presence of both *bla*_TEM_ and *bla*_CTX-M_ genes. Isolates in lanes 6–11 exhibited bands at approximately 544 bp, consistent with the detection of the *bla*_CTX-M_ gene alone.
